# Natural Products: Review for Their Effects of Anti-HBV

**DOI:** 10.1155/2020/3972390

**Published:** 2020-12-09

**Authors:** Xuqiang Liu, Changyang Ma, Zhenhua Liu, Wenyi Kang

**Affiliations:** ^1^National R & D Center for Edible Fungus Processing Technology, Henan University, Kaifeng 475004, China; ^2^Functional Food Engineering Technology Research Center, Henan Province, Kaifeng 475004, China; ^3^Joint International Research Laboratory of Food & Medicine Resource Function, Henan University, Kaifeng, 475004 Henan Province, China

## Abstract

Hepatitis B is a global infectious disease, seriously endangering human health. Currently, there are mainly interferons and nucleoside analogues treatment of hepatitis B in the clinic, which have certain therapeutic effects on hepatitis B, but their side effects and drug resistance are increasingly prominent. Therefore, it is urgently needed to discover and develop new anti-HBV drugs, especially natural products, which have novel, high efficiency, and low toxicity anti-HBV compounds with novel antiviral mechanisms. In this manuscript, the natural products (polysaccharides and 165 compounds) with the activity of antihepatitis B virus are discussed according to their chemical classes, including 14 phenylpropanoids, 8 flavonoids,12 xanthones, 13 anthroquinones, 47 terpenoids, 6 alkaloids, 15 enediynes, 11 aromatics, 18 phenylalanine dipeptides compounds, and 13 others. In addition, the anti-HBV mechanism and targets of natural product were also discussed. The aim of this review is to report new discoveries about anti-HBV natural products and to provide reference for researchers.

## 1. Introduction

**Figure 1:**
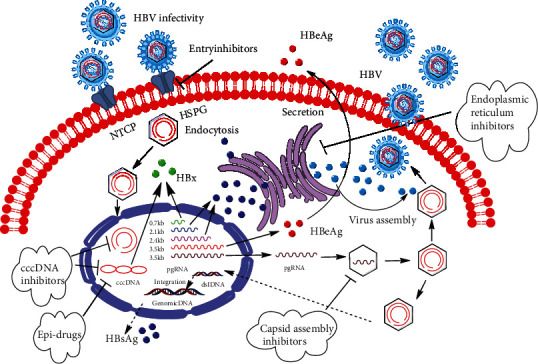
HBV life cycle and therapeutic targets. HBV life cycle: adsorption, penetration, biosynthesis, assembly, and secretion; therapeutic targets: entry inhibitors (NTCP and HSPG as the receptor-virus binding), cccDNA inhibitors (inhibiting the information of cccDNA), Epi-drugs (inhibiting the viral RNA synthesis), endoplasmic reticulum inhibitors (inhibiting the viral capsid assembly), and glucosidese inhibitors (inhibiting the secretion of HBV proteins).

Viral hepatitis B, referred to as hepatitis B, is a disease caused by the infection of hepatitis B virus (HBV, Figure 1). The infection of HBV can cause liver failure, acute or chronic hepatitis, cirrhosis, and even hepatocellular carcinoma (HCC). About 2 billion people worldwide are infected with HBV, of which 400 million are long-term carriers [1, 2]. According to research reports by the World Health Organization (WHO), about 600,000 people die of HBV infection or liver diseases related to HBV infection every year [3, 4]. China has the largest population of HBV-infected people worldwide and is confronting this large disease burden with efficient antiviral drugs.

At present, there are mainly two kinds of drugs used in the clinic, namely, interferons (INFs) with antiviral and immunoregulatory functions and nucleoside analogues that can inhibit the reverse transcription of HBV [5, 6]. In recent years, although these drugs have a certain therapeutic effect on HBV infection in the clinic, there are serious side effects and drug resistance [7, 8]. Thus, there are more and more researchers focus on natural product [9–11]. Some researches report a variety of natural medicines with novel structure and anti-HBV activity, including some candidate drugs with good anti-HBV effects. However, these reports were mainly involved in isolation and identification of compounds with anti-HBV activity; the mechanisms and targets of compounds were less. The mechanism of clinical medicines (nucleoside analogues and interferon) on anti-HBV is basically clear, but the emergence of drug-resistant HBV mutants weakens the clinical effects. Thus, the development of safe and effective anti-HBV drugs with novel mechanism is the top priority in the current research [7, 12]. In this manuscript, in order to help researchers understand HBV and develop the anti-HBV drugs, all kinds of natural products (Table 1) with anti-HBV effects and the infection process of HBV (Figure 1) [13–17] were summarized. The types of natural products with anti-HBV activity include phenylpropanoids, flavonoids, alkaloids, terpenes, glycosides, and others (such as lactones and organic acids).

## 2. The Natural Products of Anti-HBV

### 2.1. Phenylpropanoids

Phenylpropanins have a wide range of biological activities, including antitumor, antivirus, liver protection, and antioxidation. For example, a variety of lignans in fruits of *Schisandra chinensis* have liver protective effects and can reduce serum alanine aminotransferase level. Schisandrae esteril A and its analogues have been used in the treatment of chronic hepatitis in China [18].

**Figure 2:**
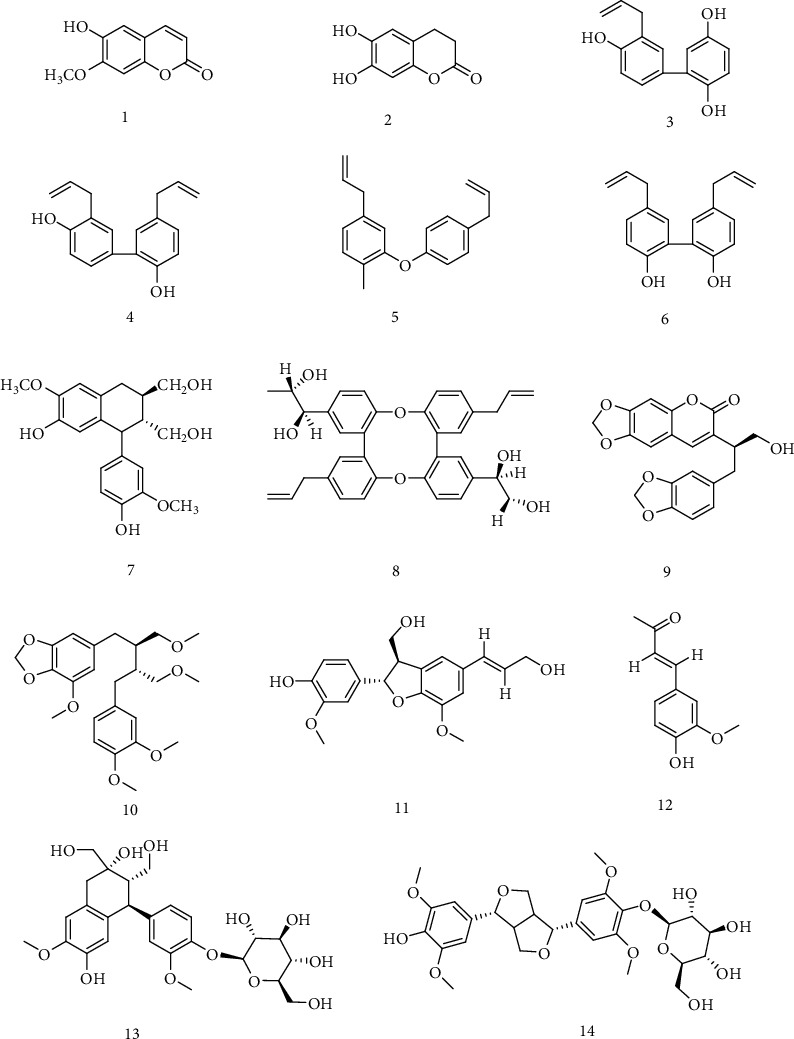
Chemical structures of representative anti-HBV phenylpropanoids **1-14**.

6-Hydroxyl-7-methoxyl-coumarin (**1**), isolated from the *Streblus asper* Lour core material, had significant anti-HBV effect on HepG 2.2.15 cells [19]. And the mechanism of compound **1** on anti-HBV effect may be related to its inhibition on secretion of hepatitis B virus surface antigen (HBsAg) and hepatitis B virus e antigen (HBeAg), and the IC_50_ were 29.60 *μ*M (selective index, SI = 6.76) and 46.41 *μ*M (SI = 4.31), respectively. Esculetin (**2**) from *Microsorium fortunei* (Moore) Ching. could not only inhibit the expression of the HBV antigens and HBV-DNA but also inhibit the expression of hepatitis B virus X(HBx) protein in a dose-dependent manner [20]. Chen et al. [21] isolated a series of phenylpropanins from the core material, bark and root of *S. asper*, all of which had significant anti-HBV activity. Among them, Magnatriol B (**3**) showed moderate anti-HBV activity by inhibiting the secretion of HBsAg and HBeAg with low cytotoxicity. Honokiol (**4**) showed significant anti-HBV activity and strong inhibition on HBsAg and HBeAg with IC_50_ of 3.14 *μ*M (SI = 21.47) and 4.74 *μ*M (SI = 14.22), respectively. The inhibition effect of honokiol on HBsAg and HBeAg was stronger than that of positive control, lamivudine. Isomagnolol (**5**) and isocarpine (**6**) from the bark and roots of *S. asper* showed significant anti-HBV activity by HepG 2.2.15 cell assay and significantly inhibited HBsAg secretion with IC_50_ of 10.34 *μ*M and 3.67 *μ*M, respectively. For inhibiting the secretion of HBeAg, IC_50_ was 8.83 *μ*M and 14.67 *μ*M, respectively, without cytotoxicity. Honokiol (**7**) and (7′*R*, 8′*S*, 7′*R*, 8′*S*)-erythron-Strebluslignanol G (**8**), isolated from methanol extract of roots of *S. asper*, have strong anti-HBV activity by inhibiting the secretion of HBsAg and HBeAg. In addition, compounds **7**and **8** could significantly inhibit the replication of HBV-DNA, with IC_50_ of 9.02 and 8.67 *μ*M, respectively [22, 23]. Coumarin lignan (**9**) isolated from the stem of *Kadsura heteroclita* could inhibit the production of HBsAg and HBeAg with concentration of 25 *μ*g/mL. The inhibition of compound **9** (57% and 48%) was even better than that of positive control, lamivudine (10% and 46%) [24]. Niranthin (**10**), isolated from *Phyllanthus niruri*, could inhibit the secretion of HBsAg and HBeAg in dose-dependent, with IC_50_ values of 16.5 *μ*M and 25.1 *μ*M. The inhibition rates of **10** on HBsAg and HBeAg were 90.4% and 83.1% with 55.5 *μ*M while the inhibition rates of lamivudine were 55.6% and 44.5% with 43.6 *μ*M. The anti-HBV effect of compound **10** was better than that of lamivudine. The inhibition rates of compound **10** on DHBV-DNA, HBsAg, and HBeAg were higher than that of lamivudine, and the recovery rate was smaller after drug withdrawal, indicating that compound **10** had a good prospect in the development of new anti-HBV drugs *in vivo* [25]. (+)-Dehydrod-iconiferyl alcohol (**11**) and dehydrozingerone (**12**) showed moderate inhibitory activities on the secretion of HBsAg with IC_50_ value of 1.94 mM (SI 1.06) and 0.50 mM (SI 2.88) [26]. (+)-Cycloolivil-4′-*O*-*β*-D-glucopyranoside (**13**) and syringaresinol 4^″^-*O*-*β*-D-glucopyranoside (**14**) showed inhibitory activity on HBsAg secretion with IC_50_ values of 0.31 ± 0.045 and 1.49 ± 0.033 mM. In particular, compound **13** exhibited inhibition not only on the secretions of HBsAg and HBeAg with IC_50_ values of 0.31 ± 0.045 mM (SI = 4.29) and 0.77 ± 0.076 mM (SI = 1.75), respectively, but also on HBV DNA replication with an IC_50_ value of 0.29 ± 0.034 mM (SI = 4.66) [27]. The chemical structures of compounds **1**~**14** showed in Figure 2.

### 2.2. Flavonoids

Flavonoids have a wide range of biological activities, including anti-inflammatory, anticancer, and antibacterial. It has a prominent role in protecting liver; for example, silymarin shows significant effect on protecting liver and has successfully developed into a protect liver medicine [28].

**Figure 3:**
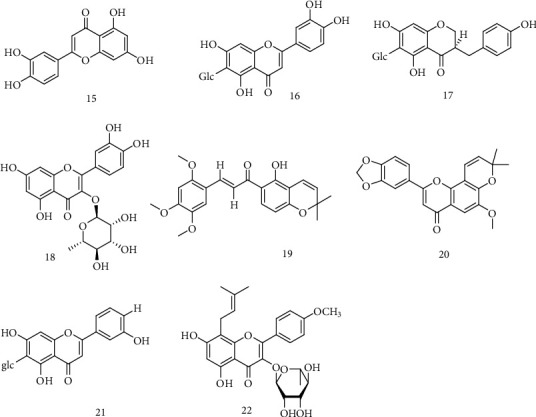
Chemical structures of representative anti-HBV flavonoids **15-22**.

Recently, flavonoids have been reported with good anti-HBV effect. Luteolin (**15**), isolated from *Swertia macrosperma* C. B. Clark, could significantly inhibit the secretion of HBsAg and HBeAg with IC_50_ values of 0.02 mM on HepG 2.2.15 cells *in vitro* [29]. Isovitexin (**16**) isolated from *S. yunnanensis* had good anti-HBV effect, which could not only inhibit the secretion of HBsAg and HBeAg, with IC_50_ values of 0.04 mM, <0.03 mM, and 0.23 mM, but also significantly inhibit the replication of HBV-DNA, with the IC_50_ values of 0.09 mM, <0.01 mM, and 0.05 mM [30]. Huang et al. [31] isolated LPRP-Et-97543 (**17**) from *Liriopemuscari* (Decne.) L.H.Bailey, which had significant anti-HBV activity and could significantly reduce the activity of Core, S, and preS promoters. Moreover, the mechanism may be that it inhibited the replication of viral DNA by regulating viral proteins. In recent years, molecular docking technology was used to screen the active ingredients against HBV, a 3D structure of HBV polymerase (Pol/RT) was modeled and docked with the active compounds, and quercetin (**18**) was proved that could enhance its anti-HBV activity up to 10% [32]. In addition, some researchers found that glabaarachalcone (**19**) and isopongachromene (**20**), isolated from *P. pinnata*, could bound with HBV-DNA polymerase protein target [33]. Isooriention (**21**), isolated from *S. mussotii*, displayed significant anti-HBV activities against the secretions of HBsAg and HBeAg with IC_50_ value of 0.79 and 1.12 mM, as well as HBV-DNA replication with IC_50_ value of 0.02 mM [34]. Epimedium Hyde II (**22**), a potential Chinese herbal active ingredient against HBV, could inhibit the replication of HBV-DNA and the expression of HBsAg and HBeAg in the serum of HBV-replicated C57BL/6 mice [35]. The chemical structures of compounds **15**~22 showed in Figure 3.

### 2.3. Xanthones

**Figure 4:**
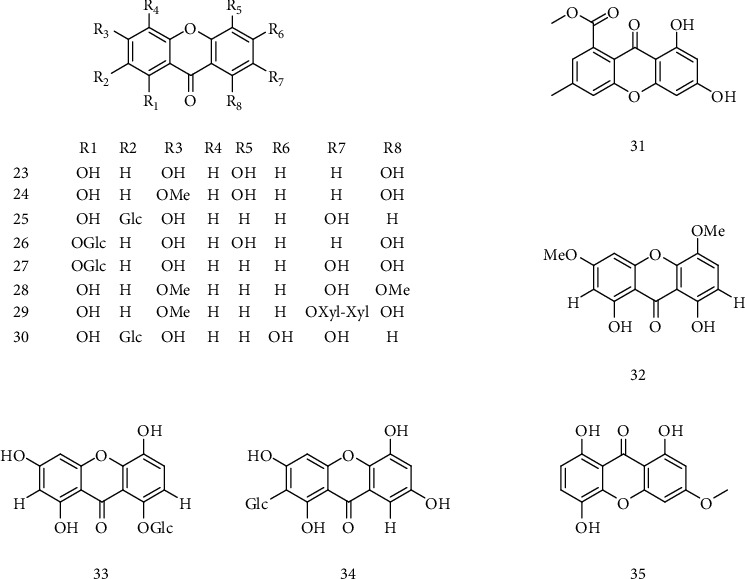
Chemical structures of representative anti-HBV xanthones **23**-**35**.

Norbellidifolin (**23**), 1,5,8-trihydroxy-3-methoxyxanthone (**24**), 2-C-*β*-D-glucopyranosyl-1,3,7-trihydroxyxanthone (**25**), norswertianolin (**26**), norswertianin-1-*O*-*β*-D-glucoside (**27**), 1,7-dihydroxy-3,8-dimethoxyxanthone (**28**), 7-*O*-[*β*-D-xylopyranosyl-(1 → 2)-*β*-D-xylopyranosyl]-1,8-dihydroxy-3-methoxyxanthone (**29**), and mangiferin (**30**) showed remarkable inhibition on HBV-DNA replication with IC_50_ values from 0.01 mM to 0.13 mM. Compounds **23**-**25** with three or more hydroxy groups showed significant inhibitory activity with IC_50_ values of 0.77, >0.98, and 0.21 mM for HBsAg, and <0.62, 0.35, and 0.04 mM for HBeAg, respectively. It was deduced that hydroxy groups in the xanthone structure were essential for maintaining the inhibitory effects on the secretion of HBsAg and HBeAg. Glycosidation of hydroxy groups led to activity decreasing against HBsAg and HBeAg by comparing the activity of compounds **24**, **26**, and **27**. It was concluded that two or more hydroxy groups were essential for inhibiting HBV-DNA replication, and methylation of hydroxy groups decreased or abolished anti-HBV activity. In addition, the position of the hydroxy groups of the isolated xanthones did not significantly affect the inhibition on HBV-DNA replication. The preliminary structure-activity relationships were deduced as (1) the anti-HBV activity of xanthones depends on the structure and substitution pattern of the hydroxy groups; (2) the hydroxy groups play very important roles in the anti-HBV activity; (3) the anti-HBV activity will be decreased after methylation orglycosidation [36]. Methyl6,8-dihydroxy-3-methyl-9-oxo-9H-xanthene-1-carboxylate (**31**), isolated from mangrove-derived aciduric fungus *Penicillium* sp., inhibited HBsAg secretion more effectively than that of the positive control, 3TC, in a dose-dependent manner [37]. 1,8-Dihydroxy-3,5-dimethoxyxanthone (**32**), norswertianolin (**33**), and neolancerin (**34**), isolated from *S. yunnanensis*, had good anti-HBV effect. Among of them, compound **34** could not only inhibit the secretion of HBsAg and HBeAg, with IC_50_ values of 0.21, 0.10, and 1.51, but also significantly inhibit the replication of HBV-DNA, with the IC_50_ values of 0.09 mM, <0.01 mM, and 0.05 mM. However, compounds **32** and **33** only showed inhibitory effect on HBV-DNA replication, which may be caused by methylation or glycoylation of the hydroxyl group of the compounds [30]. 1,5,8-Trihydroxy-3-methoxyxanthone (**35**) exhibited significant inhibitory activity on HBV-DNA replication with IC_50_ values of 0. 09 and 0. 05 m mol·L^−1^ (SI of 10. 89) and showed potent activity against the secretion of HBeAg with IC_50_ values of 0. 35 (SI of ≥2. 80) [38]. The chemical structures of compounds **23**~35 are shown in Figure 4.

### 2.4. Anthroquinones

**Figure 5:**
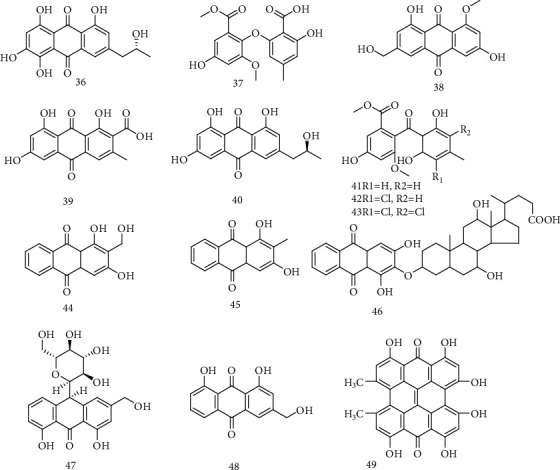
Chemical structures of representative anti-HBV anthroquinones **36-49.**

Anthroquinones, often found in the metabolites of lichens and fungi of higher plants and lower plants, have the functions of hemostasis, antisepsis, purgation, and diuretic. In recent years, the anti-HBV activity of anthroquinones was reported [39].

(−)-2′*R*-1-hydroxyisorhodoptilometrin (**36**), asterric acid (**37**), questinol (**38**), endo crocin (**39**), (+)-2′*S*-isorhodoptilometrin (**40**), sulochrin (**41**), monochlorsulochrin (**42**), and dihydrogeodin (**43**) were isolated from mangrove-derived aciduric fungus *Penicillium* sp. and inhibited HBsAg secretion more effectively than that of the positive control, 3TC, in a dose-dependent manner. Compared with 13% inhibition by 3TC, compounds **36** and **42** at 20 *μ*M inhibited HBeAg secretion by 17 and 35%, respectively. Compound **36** showed much stronger antihepatitis B virus activity than that of the positive control, lamivudine, strongly inhibiting the secretion on HBsAg and HBeAg of HepG 2.2.15 cells. These results showed that extremophiles are a valuable resource of bioactive compounds, and that pH regulation is an effective strategy to induce metabolite production in aciduric fungi [37]. Peng et al. [40] found that 1,3-dihydroxy-2-hydroxymethyl-9,10-anthraquinone (**44**), Rubiadin (**45**), and Anthraquinone bile acid conjugates (**46**) have significant anti-HBV effects on HepG2.2.15 cells. The IC_50_ values of them were 12.41, 8.03, 17.05, and 8.13 g/mL, respectively. When the drug concentrations were 8 g/mL, the inhibitory rates of HBeAg were 61.42%, 43.79%, and 69.30%, respectively. The inhibitory rates of HBsAg secreted by cells were 6.15%, 23.34%, and 43.38%, respectively. Particularly, compound **45** could not only significantly decrease HBeAg and HBsAg secretion level and inhibit HBV-DNA replication but also inhibit the proliferation of the cells and HBx protein expression in a dose-dependent manner, which might become a novel anti-HBV drug candidate. Mohammad K et al. [41] reported that anti-HBV potential of AV-derived anthroquinones, possibly via HBV-DNA polymerase inhibition for the first time. Although aloin B **(47)** exhibited novel antiviral effect, aloe-emodin (**48**) appeared as the most promising anti-HBV natural drug with CYP3A4 activating property towards its enhanced therapeutic efficacy. Lan et al. [42] found that hypericin **(49)** could significantly reduce the expression of HBV-DNA and the expression level of HBsAg and HBeAg, which was similar to lamivudine, 3TC. The chemical structures of compounds **36**~49 are shown in Figure 5.

### 2.5. Terpenoids

Terpenes are a kind of compounds with isoprene as the basic structural unit. Terpenes have extensive biological activities, mainly including anti-inflammatory and antiviral effects [43].

**Figure 6:**
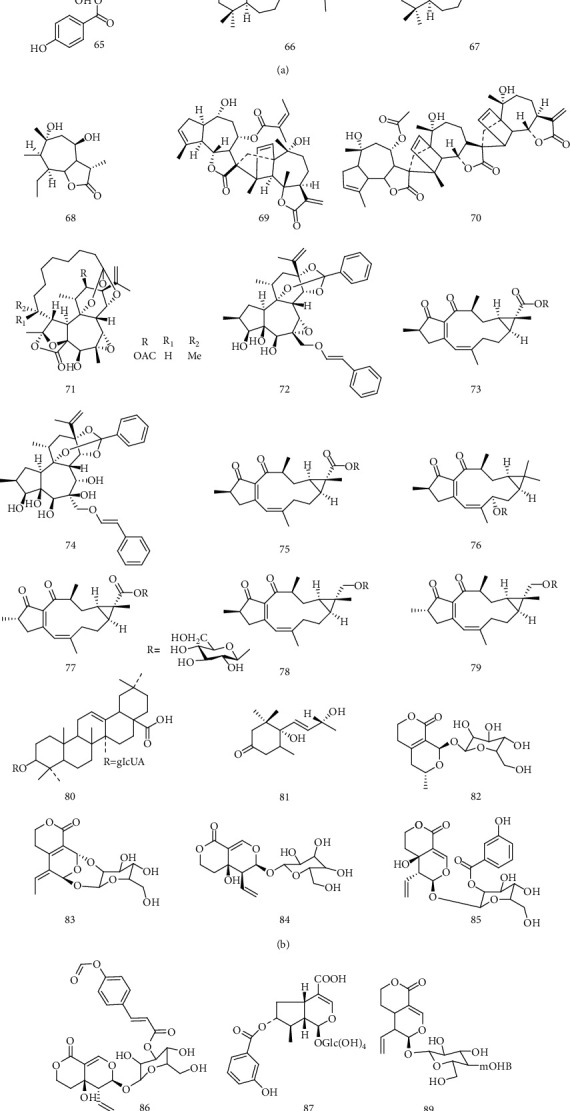
Chemical structures of representative anti-HBV terpenes **50-97**.

Li et al. [19] isolated ursolic acid (**50**) from *S. asper* core material. Compound **50** had strong anti-HBV activity by inhibiting the production of HBsAg and HBeAg, with IC_50_ of 89.91 and 97.61 *μ*M. A triterpenoid, named MH (**51**), was isolated from the *Vicia tenuifolia* Roth, which had significant inhibitory effect on the secretion of HBsAg and HBeAg in a dose-dependent manner [44]. Sweriyunnangenin A (**52**), 3-epitaraxerol (**53**), oleanolic acid (**54**), and erythrocentaurin (**55**), isolated from *S. yunnanensis*, could inhibit the secretion of HBsAg with IC_50_ values of 0.28, 0.70, and 1.26 mM, respectively. They also had good inhibitory effects on the secretion of HBeAg, with the IC_50_ values of 0.29, 1.41, and 0.94 mM, respectively. Especially, compound **55** could effectively inhibit the secretion of HBsAg and HBeAg, as well as the replication of HBV-DNA, due to its aldehyde group [26]. Zhou et al. [45] isolated a series of heptane terpenoids from the roots and rhizomes of *Aster tataricus* L. f. Among them, astataricusones B andepishionol (**56-57**) could inhibit the secretion of HBeAg, with IC_50_ value of 18.6 and 40.5 *μ*M, and the replication HBV-DNA, with IC_50_ value of 2.7 and 30.7 *μ*M. In addition, compound **56** had inhibitory effect on the secretion of HBsAg with IC_50_ value of 23.5 *μ*M. Zhou et al. [46] carried out further research on *A. tataricus*, and 6 new shionane-type triterpenes were isolated. Among them, astershionones C (**58**) had good anti-HBV activity by inhibiting the secretion of HBsAg and HBeAg and the replication of HBV-DNA with IC_50_ values of 23.0, 23.1, and 22.4 *μ*M, respectively. Bi et al. [47] found that 7 monoterpenes (4^″^-hydroxy-3^″^-methoxyalbiflorin (**59**), 6′-*O*-*p*-hydroxybenzoyl-4^″^-Hydroxyalbiflorin (**60**), albiflorin (**61**), oxypaeoniflorin (**62**), paeoniflorin (**63**),paeonins B (**64**), and benzoylpaeoniflorin (**65**)) of the *Paeonia sinjiangensis* K. Y. Pan had anti-HBV activity and could inhibit the secretion of HBsAg and HBeAg as well as the replication of HBV-DNA. Among them, compound **59** had the highest anti-HBV activity, which was even better than that of positive drug, 3TC. Perovskatone A and demethylsalvicanol (**66-67)**, isolated from *Perovskia atriplicifolia*, had anti-HBV activity. It was for the first report on the anti-HBV effect of *P. atriplicifolia* [48]. Chrysanolide B-C and A (**68-70**) were isolated from *Dendranthema indicum*, and compound **70** had unknown trimer carbon skeleton. Compounds **68-70** had good anti-HBV activity on HepG 2.2.15 cell, and their anti-HBV activity was positively correlated with the degree of polymerization [49]. In the anti-HBV test, Pimelotides A (**71**) showed significant inhibition on the secretion of HBsAg, with an IC_50_ value of 0.016 g/mL and TI up to 355.63. However, the anti-HBV mechanism of compound **71** should be carried out for the further study [50]. Genkwanine P (**73**) and laurifolioside A (**74**), isolated from *Wikstroemia chamaedaphne* Meisn, exhibited potential antihepatitis B virus activities with IC_50_ values of 46.5 and 88.3 mg/mL, respectively. Wikstroelide W (**72**), 2-epi-laurifolioside A (**75**), laurifolioside B (**76**), 2-epi-laurifolioside B (**77**),laurifolioside (**78**), and 2-epi-laurifolioside (**79**) showed certain inhibitory effects on HBV-DNA replication with the inhibition ratios ranging from 2.0% to 33.0% at the concentrations ranging from 0.39 to 6.25 mg/mL [51]. It is reported that the extracts of *Alternantheraphiloxeroides* (Mart.) Griseb have antiviral properties *in vitro*. And oleanolic acid 3-*O*-*β*-D-glucuronopyranoside (**80**) and 4,5-dihydroblumenol (**81**), isolated from the extracts, showed significant inhibition against HepG2.2.15 cells transected with cloned HBV-DNA; their inhibitive ratios were 85.38% and 87.37% at 50 *μ*g/mL, respectively [52]. Nine compounds **82-89** isolated from *S. cincta*, namely, swericinctosides A (**82**), swericinctoside B (**83**), 9-epi swertiamarin (**84**), 2′-*O*-m-hydroxybenzoyl swertiamarin (**85**), 4^″^-*O*-actyl swertianoside E (**86**), swertiaside (**87**), swertianoside C (**88**), and decentapicrin B (**89**), possessed inhibitory activity on HBV-DNA replication with IC_50_ values from 0.05 to 1.83 mM. Compounds **82**, **84**, and **86-88** showed moderate activity against HBsAg with IC_50_ values in the range of 0.24–2.46 mM, and compounds **82**, **84**, **87**, and **88** could inhibit HBV-DNA replication with IC_50_ values of 0.30–0.62 mM. Compound **87** exhibited the most promising activity against HBV-DNA replication with an IC_50_ value of 0.05 mM (SI = 29.1), as well as moderate activity against the HBsAg secretion (IC_50_ = 0.79 mM) [53]. Geng et al. [54] found that the anti-HBV activity of erythrocentaurin (ET) derivatives was significantly improved. In particular, ET derivatives 1e and 1f (**90**, **91**) showed the highest activity of inhibiting the replication of HBV-DNA, with IC_50_ values of 0.026 mM (SI > 70.8) and 0.045 mM (SI > 36.0), respectively. Swertiakoside A (**92**) and 2′-*O*-acetylswertiamarin (**93**) exhibited significant inhibitory activity on HBV-DNA replication with IC_50_values from 0.05 to 1.46 mmol·L^−1^ [38]. Huang et al. [55] isolated Asiaticoside (**94**) from *Hydrocotyle sibthorpioides* Lam and found that Asiaticoside could effectively inhibit the secretion of HBsAg and HBeAg. In addition, Asiaticoside could significantly reduce the transcription and replication of HBV-DNA by inhibiting the core, s1, s2, and x gene promoter activity. Liu et al. [56] found diosgenin (**95**) could effectively inhibit the secretion of HBsAg and HBeAg, with the inhibition rate reaching 40% and 50%. 7-Eudesm-4(15)-ene-1*β*,6*α*-diol (**96**) and Pumilaside A (**97**), isolated from *Artemisia capillaris*, exhibited promising activity against HBV-DNA replication with IC_50_ values of 19.70 and 12.01 *μ*M, with high SI values of 105.5 and 139.2. In addition, compound **97** could also suppress the secretions of HBsAg and HBeAg with the IC_50_ values of 15.02 *μ*M (SI = 111.3) and 9.00 *μ*M (SI = 185.9) [57]. The chemical structures of compounds **50**~97 showed in Figure 6.

### 2.6. Alkaloids

Alkaloids, a kind of natural nitrogen heterocyclic, have complex ring structure, most of which have physiological activity [58].

**Figure 7:**
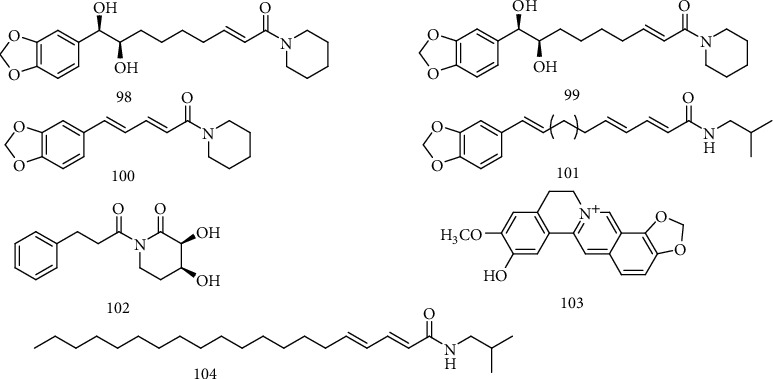
Chemical structures of representative anti-HBV alkaloids **98-104**.

Jiang et al. [59] found that the ethanol extract of *Piper longum* L. fruit had good anti-HBV effect, and erythro-1-[1-oxo-9(3,4-methylenedioxyphenyl)-8,9-dihydroxy-2*E*-nonenyl]-piperidine (**98**), threo-1-[1-oxo-9(3,4-methylenedioxyphenyl)-8,9-dihydroxy-2*E*-nonenyl]-piperidine (**99**), piperine (**100**), guineesine (**101**), and (2*E*,4*E*)-N-isobutyleicosa-2,4-dienamide (**102**) had significant inhibitory effect on the secretion of HBsAg and HBeAg on HepG 2.2.15 cells. 3*β*,4*α*-dihydroxy-1-(3-phenylpropanoyl)-piperidine-2-one (**103**), isolated from *P. longum* ethanol extract, had significant anti-HBV activity and could inhibit the secretion of HBsAg and HBeAg, with IC_50_ of 1.80 and 0.21 mM, respectively. The selectivity of compound **103** on HBeAg inhibition was up to 16.4, which was better than that of positive drug, 3TC, and has a good development prospect [60]. Zeng et al. [61] obtained a quaternary ammonium alkaloid DHCH (**104**) from *Corydalis saxicola* Bunting, which could significantly inhibit the secretion of HBsAg and HBeAg on HepG2.2.15 cells, with TI of 7.32 and 6.77, respectively. Further study showed that compound **104** could reduce the levels of cccDNA and DNA in dose and time dependence manner, with IC_50_ values of 15.08, 7.62, and 8.25 *μ*M, respectively. The chemical structures of compounds **98**~104 are shown in Figure 7.

### 2.7. Enediynes

**Figure 8:**
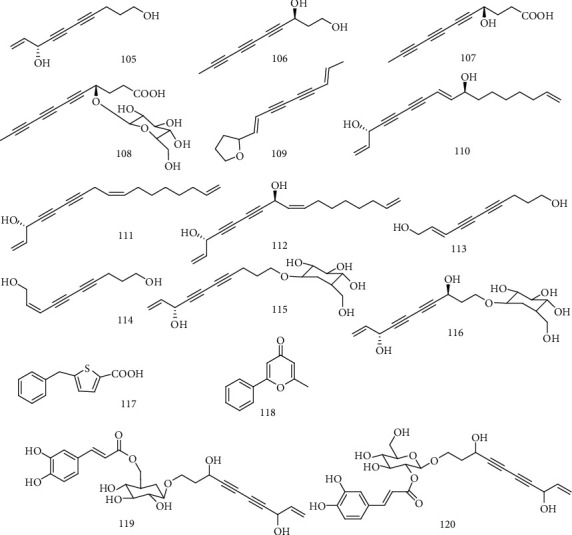
Chemical structures of representative anti-HBV enediynes **105-120**.

*A. capillaris* (Yin-Chen) is a famous traditional Chinese medicine (TCM) for treating acute and chronic hepatitis in China [62]. Geng et al. [63] isolated 14 compounds, 8*S*-deca-9-en-4,6-diyne-1,8-diol (**105**), (*S*)-deca-4,6,8-triyne-1,3-diol (**106**), (*S*)-3-hydroxyundeca-5,7,9-triynoic acid (**107**), 3*S*-Hydroxyundeca-5,9-triynoic acid 3-*O*-*β*-D-glucopyranoside (**108**), Atractylodin (**109**), Dendroarboreol B (**110**), Dehydrofalcarinol (**111**), Dehydrofalcarindiol (**112**), (*E*)-deca-2-en-4,10-diol (**113**), (*Z*)-deca-2-en-4,10-diol (**114**), 8-diol 1-*O*-*β*-D-glucopyranoside (**115**), 3*S*,8*S*-dihydroxydec-9-ene-4,6-diyne 1-*O*-*β*-D-glucopyranoside (**116**), 5-benzylthiophencarboxylic acid (**117**), and 2-methyl-6-phenyl-4H-pyran-4-one (**118**), from *A. capillaris*. All the compounds were assayed for their anti-HBV activity, and the structure-activity relationships were summarized based on the biological effects. In particular, compound **108** could significantly inhibit the secretions of HBsAg, HBeAg, and HBV-DNA replication with IC_50_ values of 197.2 (SI > 5.1), 48.7 (SI > 20.5), and 9.8 (SI > 102) *μ*M. Hydroxyl and glycosyl groups are preferable for maintaining activity. In subsequent studies, Geng et al. [64] found that 3*S*,8*S*-dihydroxydec-9-en-4,6-yne 1-*O*-(6′-O-caffeoyl)-*β*-D-glucopyranoside and 3*S*,8*S*-dihydroxydec-9-en-4,6-yne 1-*O*-(2′-*O*-caffeoyl)-*β*-D-glucopyranoside (**119-120**) had the activity against the secretions of HBsAg and HBeAg and HBV DNA replication. Especially, compounds **119** and **120** inhibited HBV-DNA replication with IC_50_ values of 0.077 ± 0.04 and 0.0127 ± 0.05 mM, with SI values of 23.6 and 17.1, respectively. Compounds **119** and **120** as a pair of isomers showed similar inhibition on HBsAg secretion with IC_50_ values of 0.797 ± 0.23 mM (SI = 2.1) and 0.887 ± 0.20 mM (SI =2.3), but no activity against HBeAg secretion. Compound **119** displayed the highest inhibitory activity on HBV-DNA replication with an IC_50_ value of 0.077 ± 0.04 mM (SI = 23.6), and compound **120** showed slightly decreased activity with an IC_50_ value of 0.127 ± 0.05 mM (SI = 17.1). The above analyses suggested that the caffeoyl group played important role in maintaining the anti-HBV activity but the substitution position may not be crucial. The chemical structures of compounds **105**~120 are shown in Figure 8.

### 2.8. Aromatics

Six phenols, *m*-hydroxybenzoic acid (**121**), *p*-hydroxybenzoic acid (**122**), *m*-hydroxy benzenmethanol (**123**), 3,4-dihydroxybenzoic acid (**124**), ethyl 3,4-dihydroxybenzoate (**125**), and ethyl 2,5-dihydroxybenzoate (**126**), exhibited anti-HBV activities by inhibiting HBsAg and HBeAg secretion with IC_50_ values from 0.23 to 5.18 mM, and HBV-DNA replication with IC_50_ values from 0.06 to 2.62 mM. Compounds **121**-**123**, with one hydroxyl and one carboxyl, showed anti-HBV activity with IC_50_ values of 3.76, 5.18, and 4.55 mM for inhibitory HBsAg secretion and 2.36, 2.54, and 2.62 mM for inhibitory HBV-DNA replication, respectively. Compounds **124**-**126** with two hydroxyls and one carboxyl displayed remarkable inhibition on HBV-DNA replication with IC_50_ values of <0.06, 0.22, and 0.29 mM. Furthermore, compounds **125** and **126** showed significant inhibitory effect on the secretion of HBsAg (IC_50_ = 0.14 and 0.23 mM) and HBeAg (IC_50_ = 5.03 and 3.74 mM) [34].

**Figure 9:**
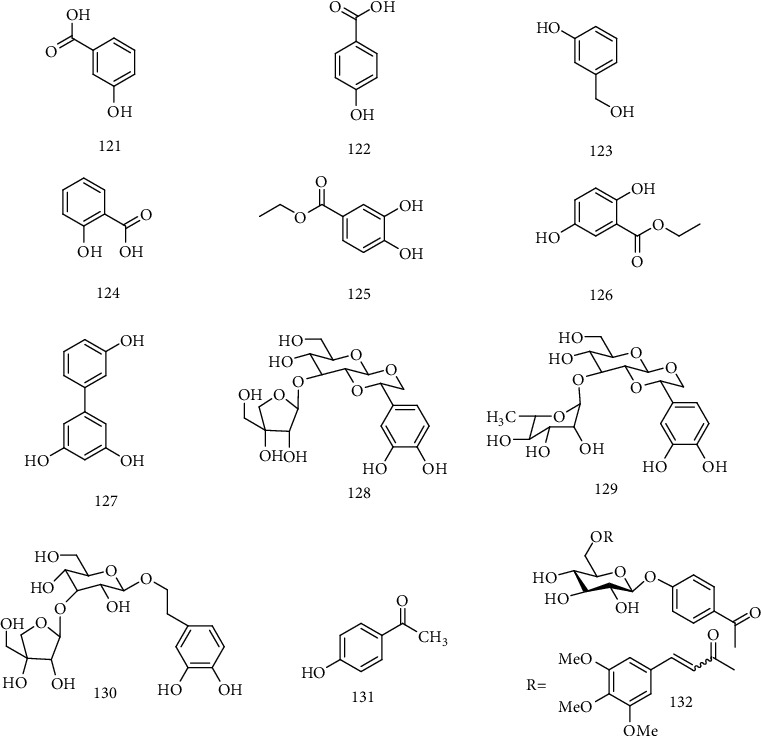
Chemical structures of representative anti-HB Varomatics **121-132**.

3,3′,5-Trihydroxybiphenyl **(127**), isolated from *S. chirayita*, showed activity against HBeAg secretion with IC _50_ values of 0.77 ± 0.076 and 5.92 ± 1.02 mM [27]. Taraffinisoside A (**128**), descaffeoyl crenatoside (**129**), and 3,4-dihydroxyphenylethanol-8-*O*-[*β*-D-apiofuranosyl (1 → 3)]-*β*-D-glucopyranoside (**130**) isolated from *Tarphochlamys affinis* (Griff.) could inhibit the secretion of HBsAg and HBeAg [65]. Huang et al. [66] isolated *p*-hydroxy acetophenone (PHAP) (**131**) from *A. morrisonensis*, which could significantly inhibit the replication of HBV. The mechanism may be that PHAP was involved in regulating the expression of surface protein genes and blocks the release of virus particles by interfering with the signaling pathway of endoplasmic reticulum. Zhao et al. [67] found that PHAP and derivatives have good anti-HBV activity, and structural modification on p-HAP and its glycoside led to a series of derivatives; among them, *p*-HAP derivative 2f (**132**) had the strongest effect on inhibiting the replication HBV-DNA (IC_50_ = 5.8 *μ*M, SI = 160.3). The primary structure-activity relationships suggested that the conjugated derivatives of *p*-HAP glycoside and substituted cinnamic acids obviously enhanced the activity against HBV-DNA replication. The chemical structures of compounds **121**~132 are shown in Figure 9.

### 2.9. Phenylalanine Dipeptides

Yang et al. [68] isolated and modified the phenylalanine dipeptide Matijin-Su (**133**) with anti-HBV activity from *Dichondra repens* Forst, and four derivatives were screened with anti-HBV activity *in vitro*. Yang et al. [69] found that compound **101** could inhibit the replication of HBV-DNA, with IC_50_ value of 1.33 *μ*M, and inhibit the replication of various mutant HBV strains. Xu et al. [70] synthesized a series of MTS derivatives with anti-HBV activity by the design of the Matijin-Su (MTS). One of the preferred MTS derivatives (Y101) was conducted in the clinical preclinical study and received the clinical approval of the CFDA.

Kuang et al. [71] used the compound MTS as lead compound; a novel MTS derivative was designed and synthesized by introducing the structure unit of veratrol acid; N-[N-(3,4-dimethoxy-benzoyl)-L-phenylalanyl]-*O*-propionyl-L-phenylalaninol (**134**), N-[N-(3,4-dimethoxy-benzoyl)-L-phenylalanyl]-4-ethoxy-L-phenylalaninol (**135**), and N-[N-(3,4-Dimethoxy-benzoyl)-L-phenylalanyl]-4-ethoxycarbonylmethyl-L-tyrosinol (**136**) were tested the anti-HBV activity *in vitro*. All the compounds have the significant anti-HBV activity. Subsequently, a series of MTS derivatives were designed and synthesized with compound MTS as the lead compound, by introducing fluorine or chlorine substitution, and the obtained MTS derivatives were tested for anti-HBV activity *in vitro*. N-[N-(4-chlorobenzoyl)-*O*-methyl-L-tyrosyl]-L-Phenylalaninol (**137**), N-[N-(4-chlorobenzoyl)-*O*-propyl-L-tyrosyl]-L-Phenylalaninol (**138**), and N-[N-(4-chlorobenzoyl)-*O*-isopropyl-L-tyrosyl]-L-Phenylalaninol (**139**) showed good anti-HBV activity, with IC_50_ of 12.61, 10.53, and 6.46 mol/L, respectively [72]. Cui et al. [73] synthesized 20 MTS derivatives containing trifluoromethyl substitution and tested the anti-HBV activity of the synthesized target compound in HepG 2.2.15 cells *in vitro*. Among them, N-[N-(3-trifluoromethylbenzoyl)-L-tyrosyl]-L-Phenylalaninol (**140**), N-[N-(3-trifluoromethylbenzoyl)-L-phenylalanyl]-*O*-propionyl-L-tyrosine methyl ester (**141**), and N-[N-(3-trifluoromethylbenzoyl)-L-phenylalanyl]-*O*-ethyl-L-tyrosine (**142**) showed strong anti-HBV activity, and their IC_50_ reached 11.74, 8.73, and 11.41 mol·L^−1^.

**Figure 10:**
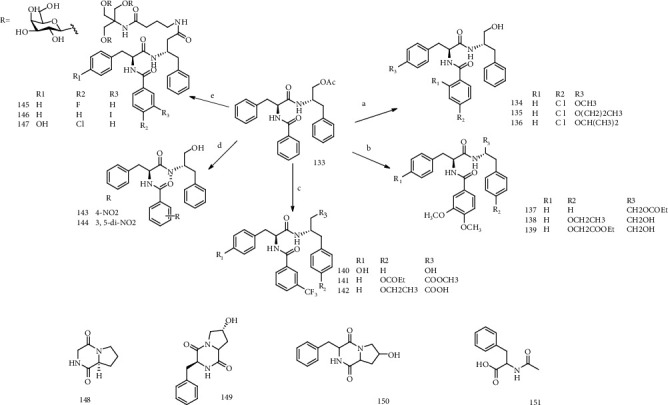
Chemical structures of representative anti-HBV phenylalanine dipeptides **133-151**.

Jang et al. [74] synthesized twenty novel *n*-methyl derivatives of MTS, among which compounds 8n (**143**) and 8o (**144**) showed certain anti-HBV activity, with IC_50_ of 52.5 mol·L^−1^ and 49.2 mol·L^−1^, respectively. Compounds 9 a-c(**145-147**) were triantennary cluster galactosides of MTS with potential for hepatic targeting. The anti-HBV activities of those were evaluated in HepG 2.2.15 cells. And all those compounds had inhibitory effect on HBV-DNA replication in HepG2 2.2.15 cells in a dose-response manner [75]. Huang et al. [76] evaluated the 20 species of marine natural small molecule compounds by HepG 2.2.15 cell lines; three kinds of compounds cyclic (glycine-L-proline) (**148**), cyclic (4-hydroxy proline-phenylalanine) (**149**), and cyclic (L-2-hydroxy proline-phenylalanine) (**150**) had anti-HBV activity on the inhibition of HBsAg, HBeAg, and HBV-DNA, with the treatment of index greater than 2. N-acetyl phenylalanine (**151**) had certain inhibitory effects on HBsAg and HBeAg with the IC_50_ values of 55.5, 69.5 *μ*g/mL, respectively [77]. The chemical structures of compounds **133-151** are shown in Figure 10.

### 2.10. Others

#### 2.10.1. Lactones

Two dimers of oxanthrone andiridoid lactone (**152, 153**) were isolated from *S. punicea*, which could inhibit the secretion of HBsAg with IC_50_ value of 0.25 and 0.29 mM, and the secretion of HBeAg with IC_50_ value of 0.86 and 0.31 mM. In addition, compounds **152**-**153** also could inhibit the replication of HBV-DNA, with the IC_50_ values of 0.18 and 0.19 mM, respectively [78]. Anislactone B (**154**), a kind of nor sesquiterpene lactone with unique structure from the fruit of *Illicium henryi*, had high anti-HBV activity and could inhibit the secretion of HBeAg on HepG 2.2.15 cell with IC_50_0.079 ± 0.035 mM*in vitro* [79].

#### 2.10.2. Isosteviol

The analogue of isosteviol, NC-8 (**155**), had anti-HBV activity by inhibiting the secretion of HBsAg and HBeAg, with the IC_50_ value of 7.89 g/mL, which was better than that of the positive control (lamivudine). The mechanism of NC-8 was interfering with HBV replication and gene expression and blocking the TLR2/NF-*κ*b signaling pathway of host cells. It is for the first report of isosteviol analogues against HBV [80]. Huang et al. [81] got a series of new derivatives, including the IN-4 (**156**) with high anti-HBV activity. The mechanism might be that IN-4 suppressed the expression of HBV gene and the replication of HBV-DNA by interfering with the NF-*κ*B signaling pathways of host cell.

#### 2.10.3. Organic Acids

**Figure 11:**
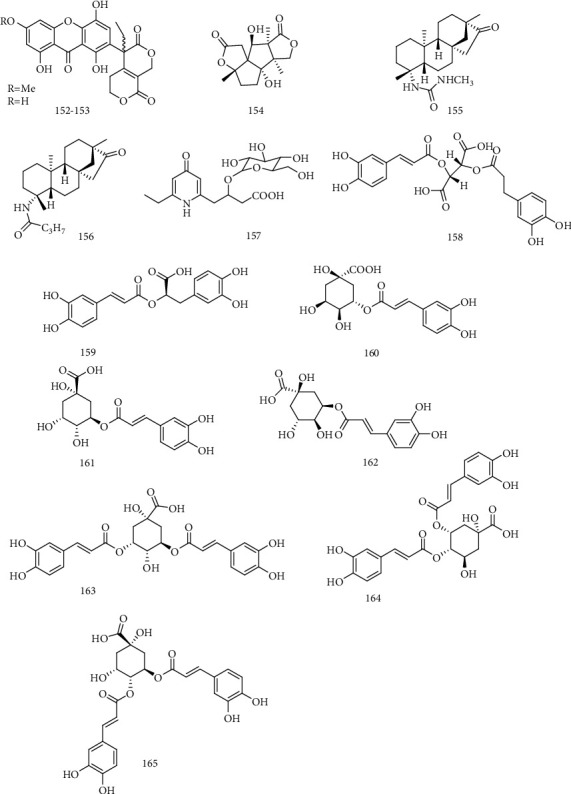
Chemical structures of representative anti-HBV compounds **152**-**165**.

Scoparamide A (**157**) could inhibit not only the secretions of HBsAg and HBeAg with IC_50_ values of 0.617 ± 0.25 mM (SI = 2.1) and 0.887 ± 0.25 mM (SI = 1.4), respectively, but also HBV-DNA replication with an IC_50_ value of 0.477 ± 0.14 mM (SI = 2.7) [64]. Zhang et al. [82] isolated cichoric acid (**158**) from the leaves of *Chicory intybus* L and found that it had significant anti-HBV activity. Rosmarinic acid (**159**) inhibits HBV replication in HBV-infected cells by specifically targeting *ε*-Pol binding. In addition, they analyzed an additional 25 rosmarinic acid derivatives and found that the “two phenolic hydroxyl groups at both ends” and the “caffeic acid-like structure” of rosmarinic acid are critical for the inhibition of *ε*-Pol binding [83]. It is well known that phenolic acids have better antiviral activity. The studies showed that 3-caffeoylquinicacid **(160)** [84] could inhibit the secretion of HBsAg, HBeAg, and the replication of HBV-DNA on Hep G 2.2.15 cells at the concentration of 100 *μ*g/mL. In order to reveal the anti-HBV activity and structure-activity relationships of the analogues of chlorogenic acid, 9 chlorogenic acid analogues were evaluated on HepG 2.2.15 cell lines *in vitro* and found that chlorogenic acid, cryptochlorogenic acid (**161**), neochlorogenic acid (**162**), 3,5-dicaffeoylquinic acid (**163**), 4,5-dicaffeoylquinic acid (**164**), and 3,4-dicaffeoylquinic acid (**165**) possessed potent activity against HBV-DNA replication with IC_50_ values in the range of 5.5 ± 0.9-13.7 ± 1.3 *μ*M. Di-caffeoyl analogues (**163-165**) also exhibited activity against the secretions of HBsAg and HBeAg. The number of caffeoyl moiety may contribute to the inhibitory activity against HBsAg and HBeAg secretions, while the position of caffeoyl units play little role on anti-HBV-DNA activities. In addition, carboxyl group is closely associated to the antiviral activity [85]. The chemical structures of compounds **152**~165 are shown in Figure 11.

#### 2.10.4. Polysaccharides

Natural polysaccharide is mainly referred to widely exists in the nature of cellulose and its derivatives, chitin, and other natural polymer materials. Polysaccharides have a wide range of biological activities, such as enhanced immunity, antiviral, and anti-inflammatory [86, 87].

In recent years, clinical researches of natural polysaccharides on anti-HBV have increased gradually; they have been proved to have significant anti-HBV effect [88]. Lentinan polysaccharide has a prominent effect on antiviral and immune regulation and is also used as an auxiliary drug for cancer and HBV [89, 90]. Zhao et al. [91] obtain two polysaccharide fractions (LEP-1 and LEP-2) from *Lentinus edodes* (Berk.) sing. They found that LEPs possess potent anti-HBV activity *in vitro*. In addition, the polysaccharides from *Hedyotis caudatifolia* Merr.et Metcalf (50, 100, and 200 mg/L) significantly inhibited the secretion and expression of HBV-DNA on HepG 2.2.15 cells and effectively inhibited the secretion of HBsAg and HBeAg. Its mechanism may be related to the activation of JAK/STAT signaling pathway and the promotion of antiviral protein expression [92]. Zhan et al. [93] found that snail polysaccharides have a certain inhibitory effect on the replication of HBV-DNA (*P* < 0.01), which indicated that the maximum inhibition rate of HBsAg and HBeAg in HepG 2.2.15 cells is 42.8% and 52.1%, respectively, slightly below the positive control group (*P* < 0.05), and the inhibition effect of snail polysaccharide on HBeAg was better than that of HBsAg. The results of real-time fluorescence quantitative PCR test showed that snail polysaccharide had a certain inhibitory effect on the replication of HBV-DNA (*P* < 0.011). The anti-HBV effect of polyporus polysaccharide may be related to the regulation of the body's immune function, breaking the body's immune tolerance or low state [94]. *Angelica sinensis* polysaccharide [95] could promote DC mature of HBV transgenic mice, raise its coordinated stimulus molecules on the surface, enhance its promoting lymphocyte proliferation and secretion, strengthen its antigen oral ability, induce cellular immune response, reduce serum concentrations of HBsAg, and play a role in antiviral immunity. Liu et al. [96] extracted Chinese whelk polysaccharide by water extraction and transfected human hepatocellular carcinoma cells with HBV-DNA cloning as an experimental model. The results showed that PCC significantly inhibited HBV-DNA in HepG 2.2.15 cells at 0.1 mg·mL^−1^ and 1 mg·mL^−1^. Xia et al. [97] investigated the effect of polysaccharides of *Sipunculus nudus* Linnaeus on anti-HBV; the results showed that polysaccharide with different dose groups were different degree of inhibition of HBV-DNA replication (*P* < 0.05), and the effects of high, middle dose group were similar to acyclovir.

## 3. Conclusion and Perspectives

At present, a variety of natural products with novel structure and high anti-HBV activity were isolated from natural resources. Among them, we found that terpenoids with antihepatitis B activity are the most (Figure 6 and Table 1), and the activity is more significant.

However, the research content were disorderly and mainly focus on the simple isolation and identification of anti-HBV activity ingredients; the in-depth studies of anti-HBV mechanisms and targets are relatively rare. Moreover, most of the studies are limited to cell level, lack of animal model experiments, and no in-depth research of ingredients with significant antihepatitis B activity. Therefore, there are three suggestions for product research and development:

### 3.1. Search for New Natural Product Resources

The research on natural products against hepatitis B mainly focuses on the field of traditional Chinese medicine on land. The research on traditional Chinese medicine against hepatitis b has been very matured. However, it is still difficult to develop active natural products against HBV. In addition, there are few researches on marine natural products, microbial fermentation products, plant polysaccharides, and other aspects. In recent years, studies have found that marine natural products have good biological activity due to their special growth environment. Huang et al. [76] screened significant anti-HBV active ingredients from small marine molecules. Microbial fermentation products are a novel source of natural products. In recent years, many novel compounds are derived from microbial fermentation products. It is an interesting way to study the anti-HBV activity of microbial fermentation products. Plant polysaccharides have a wide range of biological activities, and studies [88–95] have shown that the chemical components of polysaccharides have a good anti-HBV activity. It is of great significance to search for anti-HBV active ingredients from novel natural products.

### 3.2. Novel Method for Screening

The traditional screening of anti-HBV activity involves the separation and identification of chemical components in traditional Chinese medicinal materials and then the screening of their activity, which often takes time and effort and is difficult to obtain accurate screening results. In recent years, researchers used computer-aided drug design (molecular simulation docking) to screen out suitable compounds from the database and then carried out screening *in vitro*. This method has strong purpose and high accuracy. A series of derivatives with good anti-HBV activity were obtained by modifying the structure of known compounds with anti-HBV activity, and the derivatives with the best activity were screened out through activity test. This method also provides a new idea for discovering anti-HBV compounds with better activity [71–75].

### 3.3. Synergy Effect

Single-chemical components of natural products are no longer effective against HBV, and drug resistance will appear. For example, artemisinin is combined with other components to fight malaria. In anti-HBV studies, treatment methods of combination drugs are also widely used [98].

## Figures and Tables

**Table 1:** The compounds with anti-HBV effects from natural products.

No.	Compound	Target	Source	Ref
1	6-Hydroxyl-7-methoxyl-coumarin	HBsAg and HBeAg	*S. asper*	[19]
2	Esculetin	HBsAg, HBeAg, and HBV-DNA	*M. fortunei*	[20]
3	Magnatriol B	HBsAg and HBeAg	*S. asper*	[21]
4	Honokiol	HBsAg and HBeAg	*S. asper*	[21]
5	Isomagnolol	HBsAg	*S. asper*	[22, 23]
6	isocarpine	HBsAg	*S. asper*	[22, 23]
7	Honokiol		*S. asper*	[22, 23]
8	(7′*R*, 8′*S*, 7′*R*, 8′*S*)-erythron-Strebluslignanol G	HBsAg, HBeAg, and HBV-DNA	*S. asper*	[22, 23]
9	Coumarin lignan	HBsAg and HBeAg	*K. heteroclita*	[24]
10	Niranthin	HBsAg and HBeAg	*P. niruri*	[25]
11	(+)-Dehydrod-iconiferyl alcohol	HBsAg	*S. patens*	[26]
12	Dehydrozingerone	HBsAg	*S. patens*	[26]
13	(+)-Cycloolivil-4′-*O*-*β*-D-glucopyranoside	HBsAg, HBeAg, and HBV-DNA	*S. chirayita*	[27]
14	Syringaresinol 4-*O*-*β*-D-glucopyranoside	HBsAg	*S. chirayita*	[27]
15	Luteolin	HBsAg and HBeAg	*S. macrosperma*	[29]
16	Isovitexin	HBsAg, HBeAg, and HBV-DNA	*S. yunnanensis*	[30]
17	LPRP-Et-97543	Core, S, and preS promoters	*L. muscari*	[31]
18	Quercetin	HBeAg		[32]
19	Glabaarachalcone	HBV-DNA	*P. pinnata*	[33]
20	Isopongachromene	HBV-DNA	*P. pinnata*	[33]
21	Isooriention	HBsAg, HBeAg, and HBV-DNA	*S. mussotii*	[34]
22	Epimedium Hyde II	HBsAg, HBeAg, and HBV-DNA		[35]
23	Norbellidifolin	HBV-DNA	*S. mussotii*	[36]
24	1,5,8-Trihydroxy-3-methoxyxanthone	HBsAg and HBeAg	*S. mussotii*	[36]
25	2-C-*β*-D-glucopyranosyl-1,3,7-trihydroxyxanthone	HBsAg and HBeAg	*S. mussotii*	[36]
26	Norswertianolin	HBV-DNA	*S. mussotii*	[36]
27	Norswertianin-1-*O*-*β*-D-glucoside	HBV-DNA	*S. mussotii*	[36]
28	1,7-Dihydroxy-3,8-dimethoxyxanthone	HBV-DNA	*S. mussotii*	[36]
29	7-*O*-[*β*-D-xylopyranosyl-(1→2)-*β*-D-xylopyranosyl]-1,8-dihydroxy-3-methoxyxanthone	HBV-DNA	*S. mussotii*	[36]
30	Mangiferin	HBV-DNA	*S. mussotii*	[36]
31	Methyl6,8-dihydroxy-3-methyl-9-oxo-9H-xanthene-1-carboxylate	HBsAg	*Penicillium* sp.	[37]
32	1,8-Dihydroxy-3,5-dimethoxyxanthone	HBsAg, HBeAg, and HBV-DNA	*S. yunnanensis*	[30]
33	Norswertianolin	HBV-DNA	*S. yunnanensis*	[30]
34	Neolancerin	HBsAg, HBeAg, and HBV-DNA	*S. yunnanensis*	[30]
35	1,5,8-Trihydroxy-3-methoxyxanthone	HBeAg and HBV-DNA	*S. delavayi*	[38]
36	(−)-2′*R*-1-hydroxyisorhodoptilometrin	HBsAg and HBeAg	*Penicillium* sp.	[37]
37	Asterric acid	HBsAg	*Penicillium* sp.	[37]
38	Questinol	HBsAg	*Penicillium* sp.	[37]
39	Endocrocin	HBsAg	*Penicillium* sp.	[37]
40	(+)-2′*S*-isorhodoptilometrin	HBsAg	*Penicillium* sp.	[37]
41	Sulochrin	HBsAg	*Penicillium* sp.	[37]
42	Monochlorsulochrin	HBsAg and HBeAg	*Penicillium* sp.	[37]
43	Dihydrogeodin	HBsAg	*Penicillium* sp.	[37]
44	1,3-Dihydroxy-2-hydroxymethyl-9,10-anthraquinone	HBeAg and HBsAg	*P. connata*	[40]
45	Rubiadin	HBeAg, HBsAg, HBx, and HBV-DNA	*P. connata*	[40]
46	Anthraquinone bile acid conjugates	HBeAg and HBsAg	*P. connata*	[40]
47	Aloin B	HBV-DNA polymerase	*Aloe vera*	[41]
48	Aloe-emodin	CYP3A4	*Aloe vera*	[41]
49	Hypericin	HBsAg, HBeAg, HBV-DNA, and pgRNA		[42]
50	Ursolic acid	HBsAg and HBeAg	*S. asper*	[19]
51	MH	HBsAg and HBeAg	*V. tenuifolia*	[44]
52	Sweriyunnangenin A	HBsAg and HBeAg	*S. yunnanensis*	[26]
53	3-Epitaraxerol	HBsAg and HBeAg	*S. yunnanensis*	[26]
54	Oleanolic acid	HBsAg and HBeAg	*S. yunnanensis*	[26]
55	Erythrocentaurin	HBsAg, HBeAg, and HBV-DNA	*S. yunnanensis*	[26]
56	Astataricusones B	HBeAg, HBV-DNA, and HBsAg	*A. tataricus*	[45]
57	Epishionol	HBeAg and HBV-DNA	*A. tataricus*	[45]
58	Astershionones C	HBsAg, HBeAg, and HBV-DNA	*A. tataricus*	[46]
59	4^″^-Hydrox^″^y-3^″^-methoxyalbiflorin	HBsAg, HBeAg, and HBV-DNA	*P. sinjiangensis*	[47]
60	6′-*O*-*p*-hydroxybenzoyl-4^″^-Hydroxyalbiflorin	HBsAg, HBeAg, and HBV-DNA	*P. sinjiangensis*	[47]
61	Albiflorin	HBsAg, HBeAg, and HBV-DNA	*P. sinjiangensis*	[47]
62	Oxypaeoniflorin	HBsAg, HBeAg, and HBV-DNA	*P. sinjiangensis*	[47]
63	Paeoniflorin	HBsAg, HBeAg, and HBV-DNA	*P. sinjiangensis*	[47]
64	Paeonins B	HBsAg, HBeAg, and HBV-DNA	*P. sinjiangensis*	[47]
65	Benzoylpaeoniflorin	HBsAg, HBeAg, and HBV-DNA	*P. sinjiangensis*	[47]
66	Perovskatone A	HBsAg	*P. atriplicifolia*	[48]
67	Demethylsalvicanol	HBsAg	*P. atriplicifolia*	[48]
68	Chrysanolide B	HBsAg and HBeAg	*D. indicum*	[49]
69	Chrysanolide C	HBsAg and HBeAg	*D. indicum*	[49]
70	Chrysanolide A	HBsAg and HBeAg	*D. indicum*	[49]
71	Pimelotide A	HBsAg	*P. elongata foliage*	[50]
72	Wikstroelide W	HBV-DNA	*W. chamaedaphne*	[51]
73	Genkwanine P		*W. chamaedaphne*	[51]
74	laurifolioside A		*W. chamaedaphne*	[51]
75	2-Epi-laurifolioside A	HBV-DNA	*W. chamaedaphne*	[51]
76	Laurifolioside B	HBV-DNA	*W. chamaedaphne*	[51]
77	2-Epi-laurifolioside B	HBV-DNA	*W. chamaedaphne*	[51]
78	Laurifolioside	HBV-DNA	*W. chamaedaphne*	[51]
79	2-epi-laurifolioside	HBV-DNA	*W. chamaedaphne*	[51]
80	Oleanolic acid 3-*O*-*β*-D-glucuronopyranoside	HBV-DNA	*A.philoxeroides*	[52]
81	4,5-Dihydroblumenol	HBV-DNA	*A.philoxeroides*	[52]
82	Swericinctosides A	HBV-DNA and HBsAg	*S. cincta*	[53]
83	Swericinctoside B	HBV-DNA	*S. cincta*	[53]
84	9-Epi swertiamarin	HBV-DNA and HBsAg	*S. cincta*	[53]
85	2′-*O*-m-hydroxybenzoyl swertiamarin	HBV-DNA	*S. cincta*	[53]
86	4^″^-*O*-actyl swertianoside E	HBV-DNA and HBsAg	*S. cincta*	[53]
87	Swertiaside	HBV-DNA and HBsAg	*S. cincta*	[53]
88	Swertianoside C	HBV-DNA and HBsAg	*S. cincta*	[53]
89	Decentapicrin B	HBV-DNA	*S. cincta*	[53]
90	ET derivatives 1e	HBV-DNA	Synthesis	[54]
91	ET derivatives 1f	HBV-DNA	Synthesis	[54]
92	Swertiakoside A	HBV-DNA	*S. delavayi*	[38]
93	2′-*O*-acetylswertiamarin	HBV-DNA	*S. delavayi*	[38]
94	Asiaticoside	HBsAg, HBeAg, and HBV-DNA	*H. sibthorpioides*	[55]
95	Diosgenin	HBsAg and HBeAg		[56]
96	7-Eudesm-4(15)-ene-1*β*,6*α*-diol	HBV-DNA	*A. capillaris*	[57]
97	Pumilaside A	HBeAg, HBsAg, and HBV-DNA	*A. capillaris*	[57]
98	Erythro-1-[1-oxo-9(3,4-methylenedioxyphenyl)-8,9-dihydroxy-2*E*-nonenyl]-piperidine	HBsAg and HBeAg	*P. longum*	[59]
99	Threo-1-[1-oxo-9(3,4-methylenedioxyphenyl)-8,9-dihydroxy-2*E*-nonenyl]-piperidine	HBsAg and HBeAg	*P. longum*	[59]
100	Piperine	HBsAg and HBeAg	*P. longum*	[59]
101	Guineesine	HBsAg and HBeAg	*P. longum*	[59]
102	(2*E*,4*E*)-N-isobutyleicosa-2,4-dienamide	HBsAg and HBeAg	*P. longum*	[59]
103	3*β*,4*α*-dihydroxy-1-(3-phenylpropanoyl)-piperidine-2-one	HBsAg and HBeAg	*P. longum*	[60]
104	DHCH	HBsAg, HBeAg, cccDNA, and DNA	*C. saxicola*	[61]
105	8*S*-deca-9-en-4,6-diyne-1,8-diol	HBsAg, HBeAg, and HBV-DNA	*A. capillaris*	[63]
106	(*S*)-deca-4,6,8-triyne-1,3-diol	HBsAg, HBeAg, and HBV-DNA	*A. capillaris*	[63]
107	(*S*)-3-hydroxyundeca-5,7,9-triynoic acid	HBsAg, HBeAg, and HBV-DNA	*A. capillaris*	[63]
108	3*S*-Hydroxyundeca-5,9-triynoic acid 3-*O*-*β*-D-glucopyranoside	HBsAg, HBeAg, and HBV-DNA	*A. capillaris*	[63]
109	Atractylodin	HBsAg, HBeAg, and HBV-DNA	*A. capillaris*	[63]
110	Dendroarboreol B	HBsAg, HBeAg, and HBV-DNA	*A. capillaris*	[63]
111	Dehydrofalcarinol	HBsAg, HBeAg, and HBV-DNA	*A. capillaris*	[63]
112	Dehydrofalcarindiol	HBsAg, HBeAg, and HBV-DNA	*A. capillaris*	[63]
113	(*E*)-deca-2-en-4,10-diol	HBsAg, HBeAg, and HBV-DNA	*A. capillaris*	[63]
114	(*Z*)-deca-2-en-4,10-diol	HBsAg, HBeAg, and HBV-DNA	*A. capillaris*	[63]
115	8-Diol 1-*O*-*β*-D-glucopyranoside	HBsAg, HBeAg, and HBV-DNA	*A. capillaris*	[63]
116	3*S*,8*S*-dihydroxydec-9-ene-4,6-diyne1-*O*-*β*-D-glucopyranoside	HBsAg, HBeAg, and HBV-DNA	*A. capillaris*	[63]
117	5-Benzylthiophencarboxylic acid	HBsAg, HBeAg, and HBV-DNA	*A. capillaris*	[63]
118	2-Methyl-6-phenyl-4H-pyran-4-one	HBsAg, HBeAg, and HBV-DNA	*A. capillaris*	[63]
119	3*S*,8*S*-dihydroxydec-9-en-4,6-yne 1-*O*-(6′-*O*-caffeoyl)-*β*-D-glucopyranoside	HBsAg, HBeAg, and HBV-DNA	*A. capillaris*	[64]
120	3*S*,8*S*-dihydroxydec-9-en-4,6-yne1-*O*-(2′-*O*-caff-eoyl)-*β*-D-glucopyranoside	HBsAg, HBeAg, and HBV-DNA	*A. capillaris*	[64]
121	*m*-Hydroxybenzoic acid	HBsAg, HBeAg, and HBV-DNA	*S. mussotii*	[34]
122	*p*-Hydroxybenzoic acid	HBsAg, HBeAg, and HBV-DNA	*S. mussotii*	[34]
123	*m*-Hydroxy benzenmethanol	HBsAg, HBeAg, and HBV-DNA	*S. mussotii*	[34]
124	3,4-Dihydroxybenzoic acid	HBsAg, HBeAg, and HBV-DNA	*S. mussotii*	[34]
125	Ethyl 3,4-dihydroxybenzoate	HBsAg, HBeAg, and HBV-DNA	*S. mussotii*	[34]
126	Ethyl 2,5-dihydroxybenzoate	HBsAg, HBeAg, and HBV-DNA	*S. mussotii*	[34]
127	3,3′,5-Trihydroxybiphenyl	HBeAg	*S. chirayita*	[27]
128	TaraffinisosideA	HBsAg and HBeAg	*T. affinis*	[65]
129	Descaffeoyl crenatoside	HBsAg and HBeAg	*T. affinis*	[65]
130	3,4-Dihydroxyphenylethanol-8-*O*-[*β*-D-apiofuranosyl (1→3)]-*β*-D-glucopyranoside	HBsAg and HBeAg	*T. affinis*	[65]
131	*p*-Hydroxy acetophenone (PHAP)	HBsAg	*A. morrisonensis*	[66]
132	*p*-HAP derivative 2f	HBV-DNA	*A. capillaris*	[67]
133	Matijin-Su	HBV-DNA	*D. repens*	[68]
134	N-[N-(3,4-dimethoxy-benzoyl)-L-phenylalanyl]-*O*-propionyl-L-phenylalaninol	HBV-DNA	Synthesis	[71]
135	N-[N-(3,4-dimethoxy-benzoyl)-L-phenylalanyl]-4-ethoxy-L-phenylalaninol	HBV-DNA	Synthesis	[71]
136	N-[N-(3,4-Dimethoxy-benzoyl)-L-phenylalanyl]-4-ethoxycarbonylmethyl-L-tyrosinol	HBV-DNA	Synthesis	[71]
137	N-[N-(4-chlorobenzoyl)-*O*-methyl-L-tyrosyl]-L-Phenylalaninol	HBV-DNA	Synthesis	[72]
138	N-[N-(4-chlorobenzoyl)-*O*-propyl-L-tyrosyl]-L-Phenylalaninol	HBV-DNA	Synthesis	[72]
139	N-[N-(4-chlorobenzoyl)-*O*-isopropyl-L-tyrosyl]-L-Phenylalaninol	HBV-DNA	Synthesis	[72]
140	N-[N-(3-trifluoromethylbenzoyl)-L-tyrosyl]-L-Phenylalaninol	HBV-DNA	Synthesis	[73]
141	N-[N-(3-trifluoromethylbenzoyl)-L-phenylalanyl]-*O*-propionyl-L-tyrosine methyl ester	HBV-DNA	Synthesis	[73]
142	N-[N-(3-trifluoromethylbenzoyl)-L-phenylalanyl]-*O*-ethyl-L-tyrosine	HBV-DNA	Synthesis	[73]
143	Compound 8n	HBV-DNA	Synthesis	[74]
144	Compound 8o	HBV-DNA	Synthesis	[74]
145	Compound 9 a	HBV-DNA	Synthesis	[75]
146	Compound 9 b	HBV-DNA	Synthesis	[75]
147	Compound 9 c	HBV-DNA	Synthesis	[75]
148	Cyclic (glycine-L-proline)	HBsAg, HBeAg, and HBV-DNA		[76]
149	Cyclic (4-hydroxy proline-phenylalanine)	HBsAg, HBeAg, and HBV-DNA		[76]
150	Cyclic (L-2-hydroxy proline-phenylalanine)	HBsAg, HBeAg, and HBV-DNA		[76]
151	N-acetyl phenylalanine	HBsAg and HBeAg	*P. crinitum*	[77]
152	Two dimers of oxanthrone	HBsAg, HBeAg, and HBV-DNA	*S. punicea*	[78]
153	Iridoid lactone	HBsAg, HBeAg, and HBV-DNA	*S. punicea*	[78]
154	Anislactone B	HBeAg	*I. henryi*	[79]
155	NC-8	HBsAg and HBeAg	Synthesis	[80]
156	IN-4	HBV-DNA	Synthesis	[81]
157	Scoparamide A	HBsAg, HBeAg, and HBV-DNA	*A. scoparia*	[64]
158	Cichoric acid	DHBV-DNA	*C. intybus*	[82]
159	Rosmarinic acid	*ε*-Pol binding		[83]
160	3-Caffeoylquinicacid	HBsAg, HBeAg, and HBV-DNA	*L. japonica*	[84]
161	Cryptochlorogenic acid	HBV-DNA	*L. japonica*	[84]
162	Neochlorogenic acid	HBV-DNA	*L. japonica*	[84]
163	3,5-Dicaffeoylquinic acid	HBsAg, HBeAg, and HBV-DNA	*L. japonica*	[84]
164	4,5-Dicaffeoylquinic acid	HBsAg, HBeAg, and HBV-DNA	*L. japonica*	[84]
165	3,4-Dicaffeoylquinic acid	HBsAg, HBeAg, and HBV-DNA	*L. japonica*	[84]

## Data Availability

The data used to support the findings of this study are available from the corresponding author upon request.
